# miR-182-5p combined with brain-derived neurotrophic factor assists the diagnosis of chronic heart failure and predicts a poor prognosis

**DOI:** 10.1186/s13019-022-01802-0

**Published:** 2022-04-30

**Authors:** Fang Fang, Xiaonan Zhang, Bin Li, Shouyi Gan

**Affiliations:** 1 Department of Cardiovascular Medicine, Xianning Central Hospital, No. 228 Jingui Road, Xian’an District, Xianning City, 437000 Hubei Province China; 2 Department of Cardiovascular Medicine, The Second Affiliated Hospital of Shenyang Medical College, Xianning, 110000 Liaoning Province China

**Keywords:** miR-182-5p, Brain-derived neurotrophic factor, Chronic heart failure, Combined diagnosis, Prognosis, Receiver operating characteristic curve, Kaplan–Meier curve

## Abstract

**Objective:**

Chronic heart failure (CHF) is a general progressive disorder with high morbidity and poor prognosis. This study analyzed the serum expression and clinical value of miR-182-5p and brain-derived neurotrophic factor (BDNF) in CHF patients.

**Methods:**

A total of 82 CHF patients were selected as the study subjects (15 cases in NYHA stage I, 29 cases in stage II, 27 cases in stage III, and 11 cases in stage IV), with another 78 healthy people as the controls. The expression of serum miR-182-5p was detected by RT-qPCR. BDNF expression was measured by ELISA. Furthermore, the Pearson coefficient was used to analyze the correlation of miR-182-5p/BDNF with BNP and LVEF. ROC curve was employed to assess the potential of miR-182-5p or/and BDNF for the diagnosis of CHF. Kaplan–Meier survival curve was implemented to evaluate the prognostic value of miR-182-5p and BDNF.

**Results:**

Serum miR-182-5p level was elevated and BDNF expression was lowered in CHF patients. Serum miR-182-5p in CHF patients was positively-related with BNP and inversely-correlated with LVEF, while serum BDNF was negatively-linked with BNP and positively-correlated with LVEF. ROC curve indicated the diagnostic value of serum miR-182-5p and BDNF for CHF and the diagnostic accuracy of miR-182-5p combined with BDNF was improved. Kaplan–Meier analysis unveiled that miR-182-5p low expression and BDNF high expression could predict the overall survival in CHF patients.

**Conclusion:**

miR-182-5p expression is increased and BDNF level is decreased in CHF patients. miR-182-5p combined with BDNF can assist the diagnosis of CHF and predict a poor prognosis.

## Introduction

Chronic heart failure (CHF) refers to a collection of complicated clinical syndromes resulting from aberrant changes in cardiac function and structure [1]. The prevalence of HF in western countries is appropriately 1–2% with above 10% among people over 70 years old and about 5–10 in 1000 people suffer from HF annually, which is the major reason for outpatient visits [2]. The contributors to CHF consist of common risk factors such as hypertension, ischemic injury and metabolic syndrome, immune-related causes such as autoimmune reactions and infections (bacterial and viral), mechanical changes including valve and dysfunction, as well as genetic heart disease [3]. CHF is featured by reduced exercise capacity, unpredictable trajectory, lowered quality of life, and high symptom burden [4–6]. The symptoms include fatigue, breathlessness, and objective clinical signs including pulmonary rales, pleural effusion, tachycardia, tachypnea, fluid retention, and ankle swelling [7]. The goal of HF treatment is not only to alleviate symptoms and improve life quality but also to block the progression of cardiac remodeling and decrease hospitalization and death of HF patients [8]. Although pharmacotherapy exerts some improvement, the mortality and morbidity among the community population with CHF are still high [9], and the prognosis is poor [10, 11]. Hence, it is urgent to explore the new and effective biomarkers for the diagnosis, treatment, and prognosis of CHF.

microRNAs (miRNAs) are short, endogenous, non-coding single-stranded RNA sequences (with a length of 19–24 nucleotides) that act as gene modulators at a post-transcriptional level by binding to the 3′-untranslated regions of target mRNAs, thus leading to the degradation and translational suppression of mRNAs [12, 13]. miRNAs play vital roles in regulating the complex biological processes of different cardiovascular diseases, such as HF, arrhythmias, ischemic heart disease, and left ventricular hypertrophy [14]. Plentiful circulating miRNAs are regarded as potential indicators of cardiac diseases, including HF and myocardial infarction [15]. miR-182-5p, a member of the miR-183/96/182 cluster, is related to heart disease [16, 17]. miR-182 is upregulated in congestive HF [18]. However, the role of miR-182-5p in CHF diagnosis and prognosis remains unknown and needs to be investigated.

Brain-derived neurotrophic factor (BDNF) regulates neurotrophin and neuronal survival in the brain, exerting important functions in the survival, growth, differentiation, and repair of cells, synaptic plasticity, and adult/embryonic neurogenesis in the neurons of the central nervous system by stimulating tropomyosin receptor kinase B [19, 20]. BDNF is a pivotal player in the progression of the cardiovascular system and a low circulating BDNF level is linked with higher cardiovascular risks [21, 22]. Moreover, the level of serum BDNF is reduced in HF patients [23], indicating the correlation between BDNF and CHF. Nonetheless, there are very few reports on the significance of miR-182-5p and BDNF levels on the diagnosis and prognosis of CHF. Therefore, this study assessed the effect of serum miR-182-5p and BDNF expressions in diagnosing CHF, judging severity, and predicting prognosis.

## Methods

### Ethics statement

This study was approved by the ethics committee of Xianning Central Hospital (Approval number: K2020-016). The informed consent was signed by all participants.

### Study subjects

A total of 82 CHF patients diagnosed and treated in Xianning Central Hospital from January 2017 to March 2018 were included in the study group. The diagnosis of CHF was based on the *European Society of Cardiology (ESC) guidelines for the diagnosis and treatment of acute and chronic heart failure 2012*. In addition, non-CHF subjects with the matched age and gender in the same period who received health examinations were selected as the control group.

### Inclusion and exclusion criteria

Inclusion criteria were: (1) diagnosed with HF for more than half a year, in line with the diagnostic criteria for CHF [24]; (2) with complete clinical data; (3) with HF treatment for 1 month or more after this study; (4) over 60 years old.

Exclusion criteria were: (1) with CHF caused by other factors, including rheumatic heart disease and primary dilated cardiomyopathy; (2) suffered from a history of mental illness, liver and kidney failure; (3) with incomplete examination data; (4) with malignant tumors.

Cardiac function was classified according to the New York Heart Association (NYHA) classification system revised in 1928 and 1994 [25]. Stage I: patients suffering from heart disease but without limitation to daily activities, and the normal activities did not cause fatigue, palpitation, dyspnea, and angina pectoris. Stage II: patient with heart disease had a slight limitation of activities after normal work but the symptom disappeared after a short rest, and daily activities could cause the symptoms including mild dyspnea, palpitation, angina pectoris, and fatigue caused. Stage III: heart disease patients had a significant limitation to daily activities and a range of symptoms caused by the activities below the daily level. Stage IV: heart disease patients could not perform any mild physical activity, and symptoms of HF might appear even at rest, with the mild physical activity aggravating the HF.

### Data collection and follow-up

The baseline data of age, gender, smoking, total cholesterol (TC), triglyceride (TG), low-density lipoprotein cholesterol (LDL-C), high-density lipoprotein cholesterol (HDL-C), uric acid (UA), brain natriuretic peptide (BNP), left ventricle ejection fraction (LVEF), and the complications (including hypertension and diabetes), as well as NYHA stage of cardiac function in patients, were recorded. Venous blood (3 mL) was collected from the enrolled CHF patients the next morning after admission. Blood samples were also collected from control subjects and stored at room temperature for 30 min followed by centrifugation at 2800×*g* for 10 min. Later, the separated supernatant was stored in a −80 °C freezer.

The patients received conventional treatment and were orally administered with enalapril maleate tablets, 10 mg/time and 1 time/day; metoprolol sustained-release tablets (Southwest Pharmaceutical Co., Ltd., Chongqing, China), with the initial dose of 6.25 mg/time and 2 times/day, and then doubled weekly until the maximum dose reached 100 mg/time, but the maximum daily dose not exceeding 400 mg; spironolactone tablets, 20 mg/time and 1 time/day (Beijing zhongxin pharmaceutical factory, Beijing, China). All patients received telephone follow-up every 2 months for 24 months. The survival status of all patients was collected and analyzed, and the death of CHF patients and the cause of death were recorded.

### Reverse transcription quantitative polymerase chain reaction (RT-qPCR)

RT-qPCR was employed to determine the expression of miR-182-5p in the serum samples of the study population. Total RNA was extracted from serum samples using TRIzol reagent (Thermo Fisher Scientific, Waltham, MA, USA), and the total RNA was separated using mirVana PARIS kits (Thermo Fisher Scientific). cDNA was synthesized via PrimeScript RT reagent kit (TaKaRa, Otsu, Shiga, Japan). The extracted RNA concentration and purity were determined by an ultraviolet spectrophotometer. RT-qPCR reactions were performed using ChamQTM SYBR qPCR Master Mix (Vazyme, Nanjing, China) with the following reaction conditions: 95 °C for 30 s, then 40 cycles of 95 °C for 10 s and 60 °C for 30 s. The relative expression of miR-182-5p after normalization to the internal reference U6 was calculated using the 2^−ΔΔCt^ method. Primer sequences are presented in Table 1.

**Table 1:** Primer sequences

Gene	Forward 5′-3′	Reverse 5′-3′
miR-182-5p	ATCACTTTTGGCAATGGTAGAACT	TATGGTTTTGACGACTGTGTGAT
U6	GCTTCGGCAGCACATATACTAA	AACGCTTCACGAATTTGCGT

miR-182-5p, microRNA-182-5p

### Enzyme-linked immunosorbent assay (ELISA)

Serum BDNF was measured using human BDNF ELISA kit (cat no. KE00096, ProteinTech Group, Inc., Rosemont, IL, USA). The specific BDNF antibody was pre-coated on a 96-well microplate, and the wells were respectively added with standard and test samples, with the blank well set up meanwhile. After adding the biotinylated BDNF antibody, the wells were rinsed thoroughly to remove the unbound biotinylated antibody. Next, the wells were supplemented with horseradish peroxidase-labeled avidin and then washed again followed by adding the 3,3′,5,5′-tetramethylbenzidine (TMB) substrate (ProteinTech Group, Inc.) for color development. Later, TMB turned blue in catalysis and turned yellow under acid action. The optical density value was determined using ELISA at a wavelength of 450 nm, with the corresponding concentration converted using the standard curve.

### Dual-luciferase reporter assay

H9C2 cardiomyocytes purchased from Procell (Wuhan, China) were seeded into 24-well plates at 1 × 10^5^ cells/well. The binding sites of BDNF and miR-182-5p were firstly identified using the bioinformatics software Starbase (http://starbase.sysu.edu.cn/index.php). According to the prediction results, the complementary binding sequences of miR-182-5p to BDNF and the mutant sequences were amplified and cloned into the pmiR-GLO luciferase vector (Promega) to construct the BDNF-wild type (WT) plasmid and the corresponding BDNF-mutant (MUT) plasmid. Subsequently, miR-182-5p mimics or miR-182-5p negative control (RiboBio, Guangzhou, China) and reporter plasmids were co-transfected into H9C2 cells using Lipofectamine 2000 (Invitrogen, Carlsbad, CA, USA) respectively. After 48 h of transfection, luciferase activity was measured using a dual-luciferase assay kit (Promega, Madison, WI, USA) in line with the instructions.

### Data analysis

The statistical analysis and plotting of data were implemented using SPSS 21.0 statistical software (IBM Corp. Armonk, NY, USA) and GraphPad Prism 6.0 software (GraphPad Software Inc., San Diego, CA, USA). The normal distribution was verified by Shapiro–Wilk test. Measurement data of normal distribution were exhibited as mean ± standard deviation (SD) and analyzed by independent sample *t* test for comparisons between groups. Measurement data of non-normal distribution were expressed as quartile and analyzed by Mann–Whitney U test for comparisons between groups. Enumeration data were displayed as cases and percentages, and analyzed by Chi-square test for comparisons between groups. Receiver operating characteristic (ROC) curve was employed to assess the diagnostic efficacy of parameters and obtain the cutoff values. The effect of miR-182-5p expression on the incidence of poor prognosis was analyzed using the Chi-square test and Kaplan–Meier method, with Log rank assay testing the group differences of Kaplan–Meier curves. *P* value was obtained from a two-sided test. The *P* < 0.05 and *P* < 0.01 indicated statistical significance.

## Results

### Clinical baseline features of participants

This study recruited 82 CHF patients and 78 controls. There were 45 males and 37 females with a mean age of 64.93 ± 1.44 years in CHF patients, and 41 males and 37 females with a mean age of 65.44 ± 1.41 years in the control group. The basic information of enrolled participants was shown in Table 2. The results revealed that the two groups showed no differences in age, gender, BMI, smoking history, drinking history, TC, TG, LDL-C, HDL-C, or UA. Moreover, in terms of complications, there also existed no differences in hypertension and diabetes. However, BNP expression was significantly higher and LVEF level was evidently lower in the CHF group than those in the control group (all *P* < 0.001).

**Table 2:** Comparisons of clinical baseline features

Feature	Controls	CHF	*P* value
	(N = 78)	(N = 82)	
Age (year)	65.44 ± 1.41	64.93 ± 1.44	0.887
Gender (male/female)	41/37	45/37	0.770
BMI (kg/m^2^)	24.96 ± 0.325	25.07 ± 0.345	0.988
Smoking history (never/ever)	61/17	68/14	0.453
Drinking history (never/ever)	54/24	57/25	0.969
TC (nM)	4.65 ± 0.18	4.62 ± 0.17	0.282
TG (nM)	1.34 ± 0.63	1.42 ± 0.66	0.439
LDL-C (nM)	2.93 ± 0.15	3.05 ± 0.13	0.865
HDL-C (nM)	1.17 ± 0.33	1.18 ± 0.05	0.781
UA (µM)	355.23 ± 14.95	352.38 ± 13.51	0.207
BNP (ng/l)	66.57 ± 20.93	1564.595 ± 875.43	< 0.001
LVEF (%)	58.76 ± 0.64	29.93 ± 3.52	< 0.001
Complication (no/yes)			
Hypertension	44/34	57/25	0.0870
Diabetes	47/31	53/29	0.5704
COPD	–	69/13	–
Anemia	–		–
Fluid and electrolyte imbalance	–	45/37	–
NYHA stage			
I	–	15	–
II	–	29	–
III	–	27	–
IV	–	11	–

Data were expressed as mean ± standard deviation or N

CHF, chronic heart failure; BMI, body mass index; TC, total cholesterol; TG, triglyceride; LDL-C, low-density lipoprotein cholesterol; HDL-C, high-density lipoprotein cholesterol; UA, uric acid; BNP, brain natriuretic peptide; LVEF, left ventricle ejection fraction; COPD, chronic obstructive pulmonary disease; NYHA, New York Heart Association

### miR-182-5p was upregulated in the serum of CHF patients

**Fig. 1:**
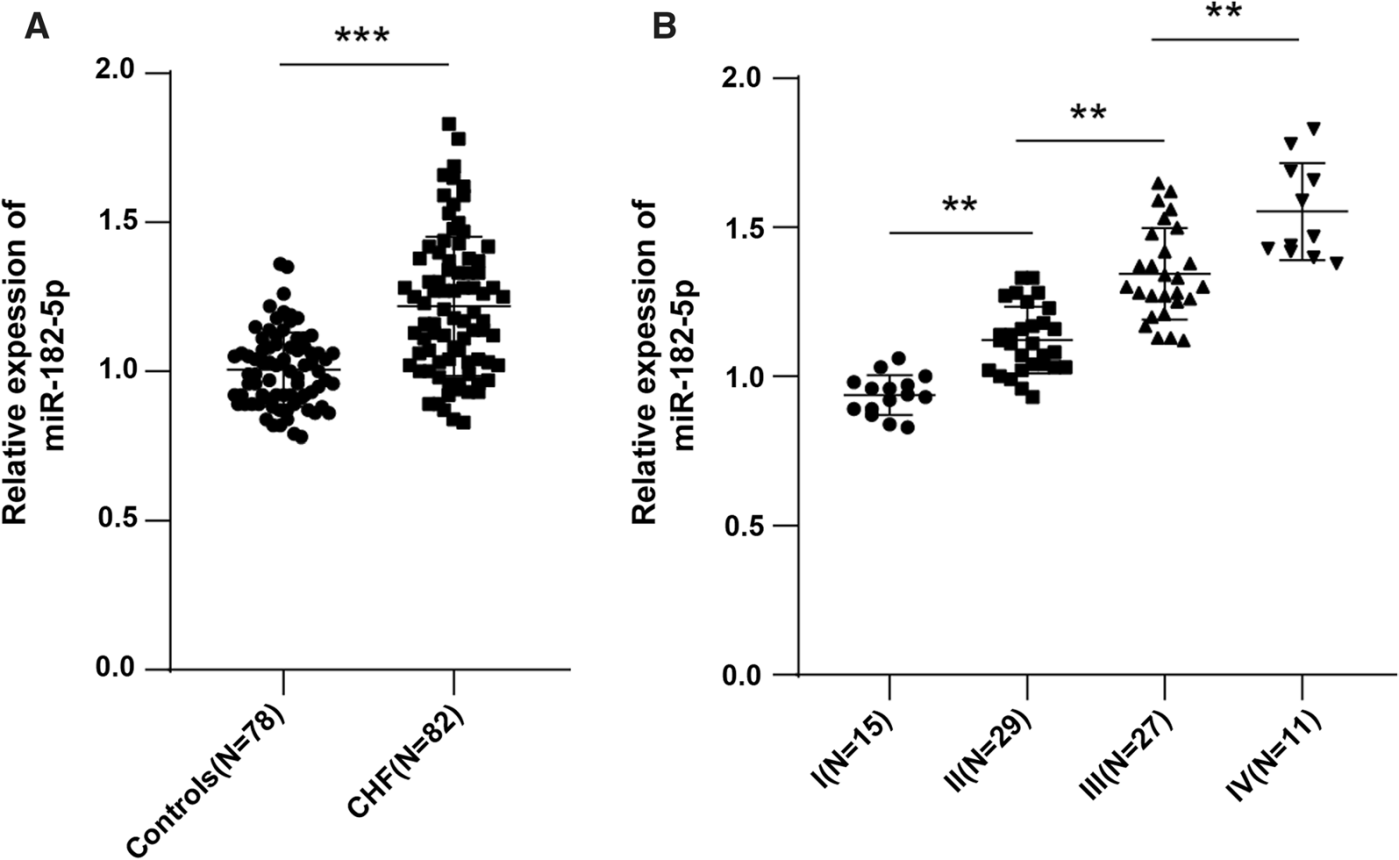
miR-182-5p was upregulated in the serum of CHF patients. RT-qPCR was used to detect: **A** the expression of miR-182-5p in the serum of CHF patients and controls; **B** miR-182-5p expression in the serum of CHF patients with different NYHA functional stages. Data were as mean ± SD and independent unpaired *t* test was adopted for comparisons between two groups. **P < 0.01, ***P < 0.001

The expression of miR-182-5p in the serum of CHF patients and controls was detected by RT-qPCR. The results suggested that the miR-182-5p expression in the serum of CHF patients was markedly higher than that of controls (*P* < 0.01) (Fig. 1A). In addition, the miR-182-5p level in the serum of patients with different NYHA stages was compared, and the results indicated an elevation of miR-182-5p in parallel with the increase of NYHA stage of cardiac function (all *P* < 0.01) (Fig. 1B).

### BDNF was downregulated in the serum of CHF patients and negatively correlated with miR-182-5p

**Fig. 2:**
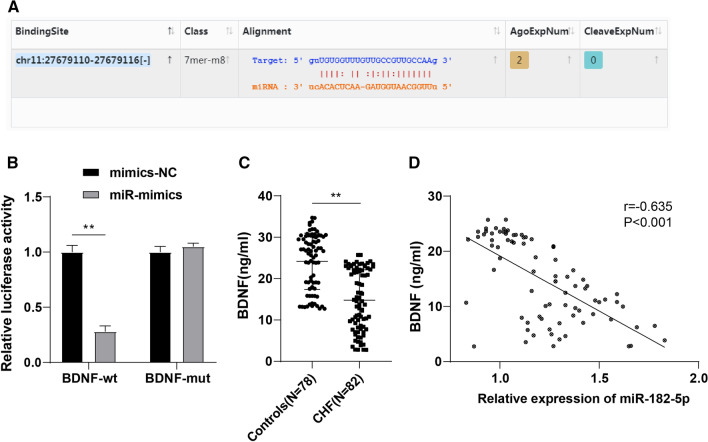
Analysis of BDNF expression and its correlation with miR-182-5p in CHF patients. **A** The binding sites of miR-182-5p to BDNF were predicted by Starbase database; **B** The targeted binding of miR-182-5p and BDNF was validated using dual-luciferase assay; **C** BDNF expression in the serum of CHF patients and controls was determined via ELISA; **D** The correlation between miR-182-5p and BDNF in CHF patients was analyzed by Pearson assay. Data were expressed as mean ± SD and independent unpaired *t* test was used for comparisons between two groups. **P < 0.01

Studies have reported that BDNF deficiency impairs the survival of intramyocardial arterial and capillary endothelial cells [26], and it is involved in the maintenance of baroreflex sensitivity in CHF patients, affecting the progression and poor prognosis of CHF [27, 28]. Starbase database predicted the binding sites between miR-182-5p and BDNF (Fig. 2A), and a dual-luciferase assay verified the targeted binding of miR-182-5p to BDNF in H9C2 cardiomyocytes (Fig. 2B). Furthermore, the serum expression of BDNF in CHF patients and controls was measured using ELISA kits, which demonstrated remarkably lowered serum BDNF expression in CHF patients relative to the controls (*P* < 0.01) (Fig. 2C). Moreover, the Pearson method was used to analyze the correlation between miR-182-5p and BDNF, and the results unveiled the prominently inverse association of miR-182-5p expression and BDNF level in the serum of CHF patients (r = −0.635, *P* < 0.001) (Fig. 2D).

### Correlation analysis of serum miR-182-5p/BDNF with BNP and LVEF in CHF patients

**Fig. 3:**
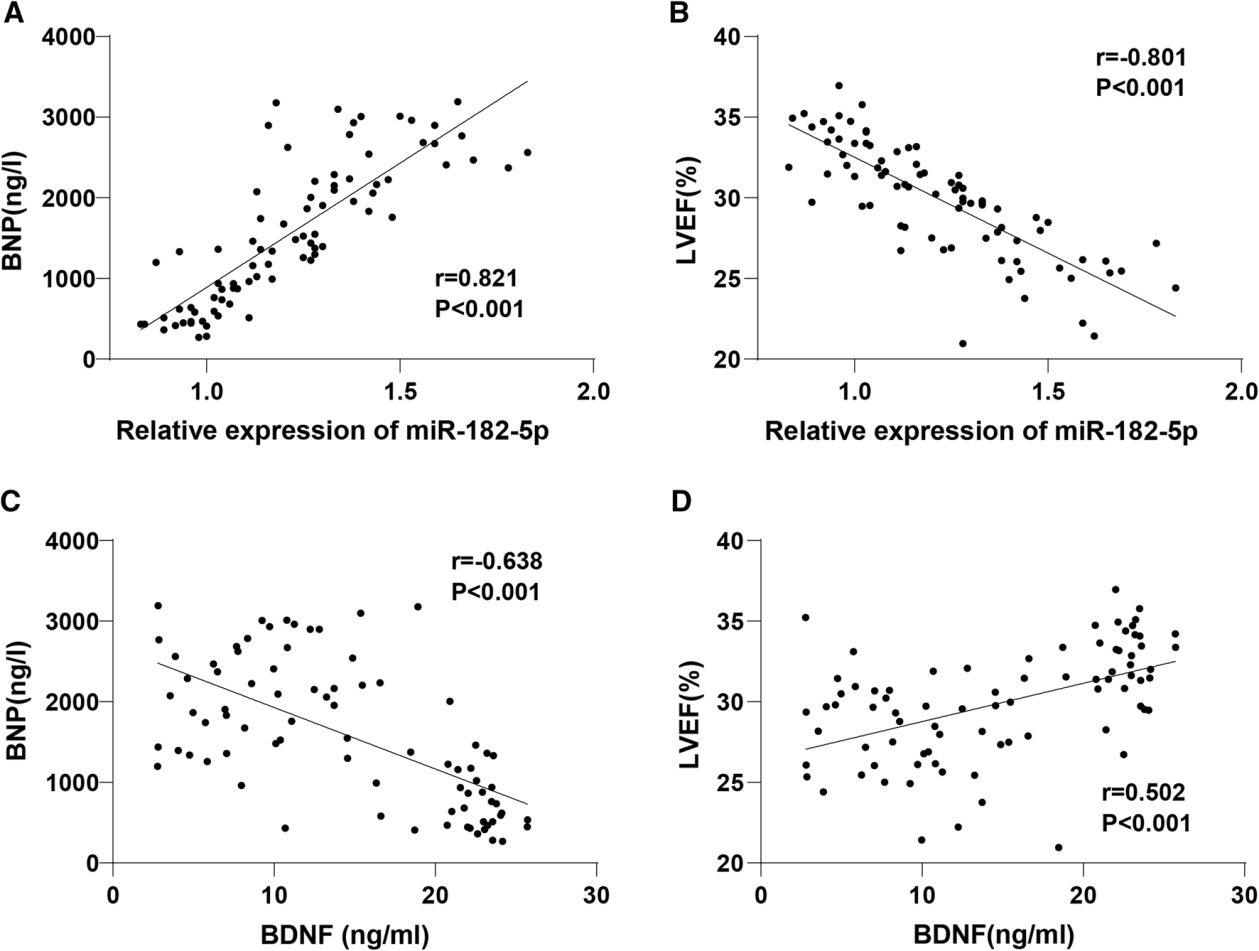
Correlation analysis of serum miR-182-5p/BDNF with BNP and LVEF in CHF patients. In CHF patients, the Pearson method was employed to analyze: **A** the correlation of miR-182-5p and BDNF; **B** the association between miR-182-5p and LVEF; **C** the relevance of BDNF and BNP; **D** the relation between BDNF and LVEF

Studies have revealed that BNP and LVEF are increasingly used for CHF diagnosis and evaluation of prognosis [29, 30]. We had revealed that BNP level was significantly raised and LVEF level was clearly diminished in the serum of CHF patients compared to the controls. To further investigate the clinical value of miR-182-5p expression in CHF patients, we analyzed the correlation of miR-182-5p/BDNF with BNP and LVEF by the Pearson method. The results showed that the serum miR-182-5p level in CHF patients was remarkably positively related with BNP (r = 0.821, *P* < 0.001) (Fig. 3A) and inversely correlated with LVEF (r = −0.801, *P* < 0.001) (Fig. 3B). The serum expression of BDNF in CHF patients was negatively linked with BNP (r = −0.638, *P* < 0.001) (Fig. 3C) and positively correlated with LVEF (r = 0.502, *P* < 0.001) (Fig. 3D).

### miR-182-5p combined with BDNF had a high diagnostic efficacy for CHF

**Fig. 4:**
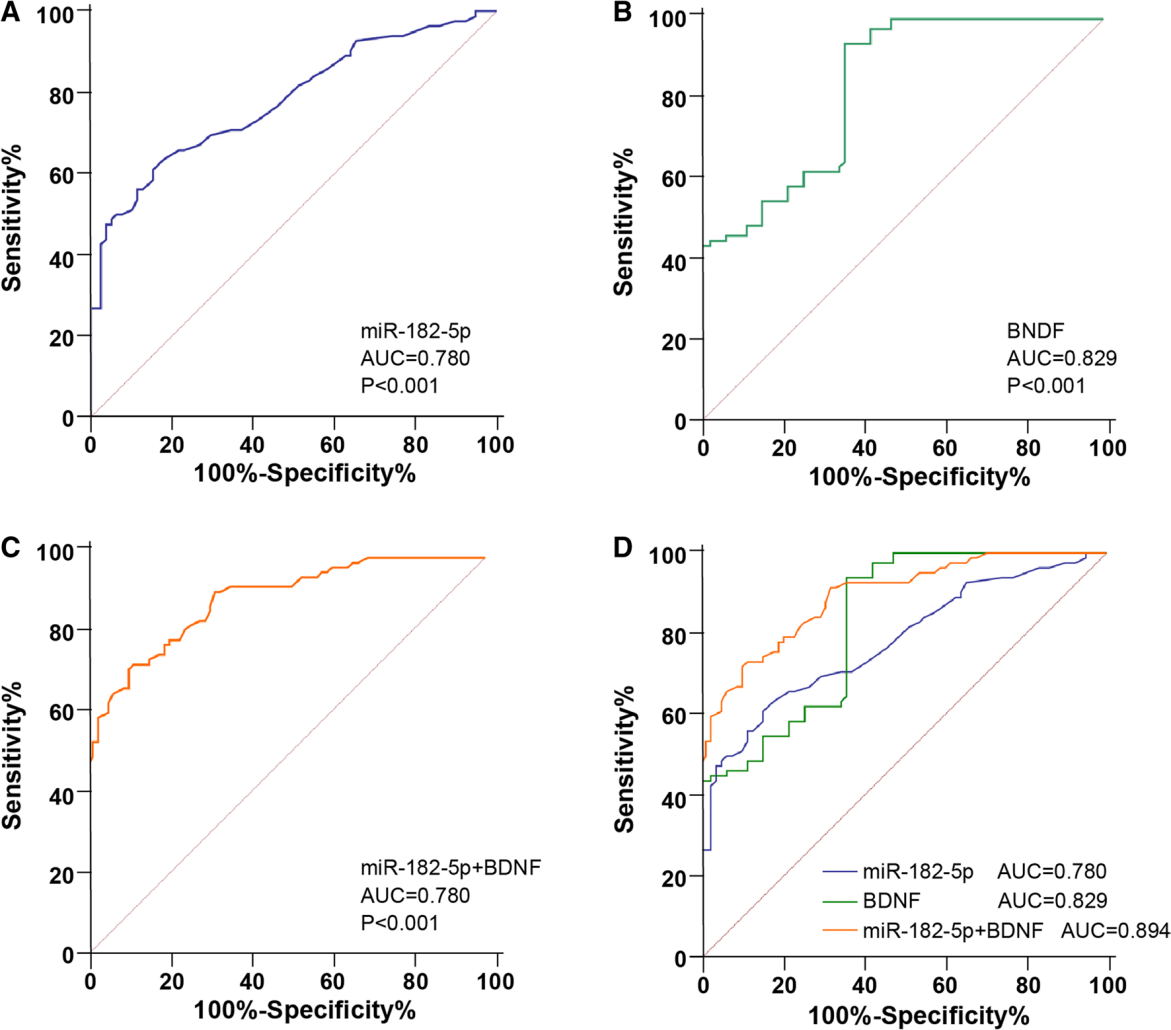
miR-182-5p combined with BDNF had high diagnostic efficacy for CHF. ROC curve was plotted to analyze the diagnostic efficacy for CHF of: **A** serum miR-182-5p; **B** serum BDNF; **C** miR-182-5p combined with BDNF; **D** MedCalc assay was employed to analyze the AUC differences

To further explore the clinical diagnostic significance of serum miR-182-5p expression for CHF patients, we assessed the diagnostic efficacy of miR-182-5p, BDNF alone, and miR-182-5p combined with BDNF for CHF by ROC curve. The analysis revealed that in the diagnosis of CHF, the area under the curve (AUC) of miR-182-5p was 0.780 and the cutoff value was 1.125, with 60.89% sensitivity and 84.62% specificity (Fig. 4A), and the AUC of BDNF was 0.829 and the cutoff value was 23.70, with 93.90% sensitivity and 64.10% specificity (Fig. 4B). The AUC of miR-182-5p combined with BDNF in diagnosing CHF was 0.894 and the cutoff value was 0.665, with 71.95% sensitivity and 89.74% specificity (Fig. 4C). MedCalc assay used for analyzing the AUC differences illustrated that the diagnostic value of miR-182-5p combined with BDNF was prominently higher than that of miR-182-5p or BDNF alone (all *P* < 0.05) (Fig. 4D). Altogether, miR-182-5p combined with BDNF had a high diagnostic efficacy for CHF.

### Analysis of the prognostic value of miR-182-5p and BDNF for CHF

**Fig. 5:**
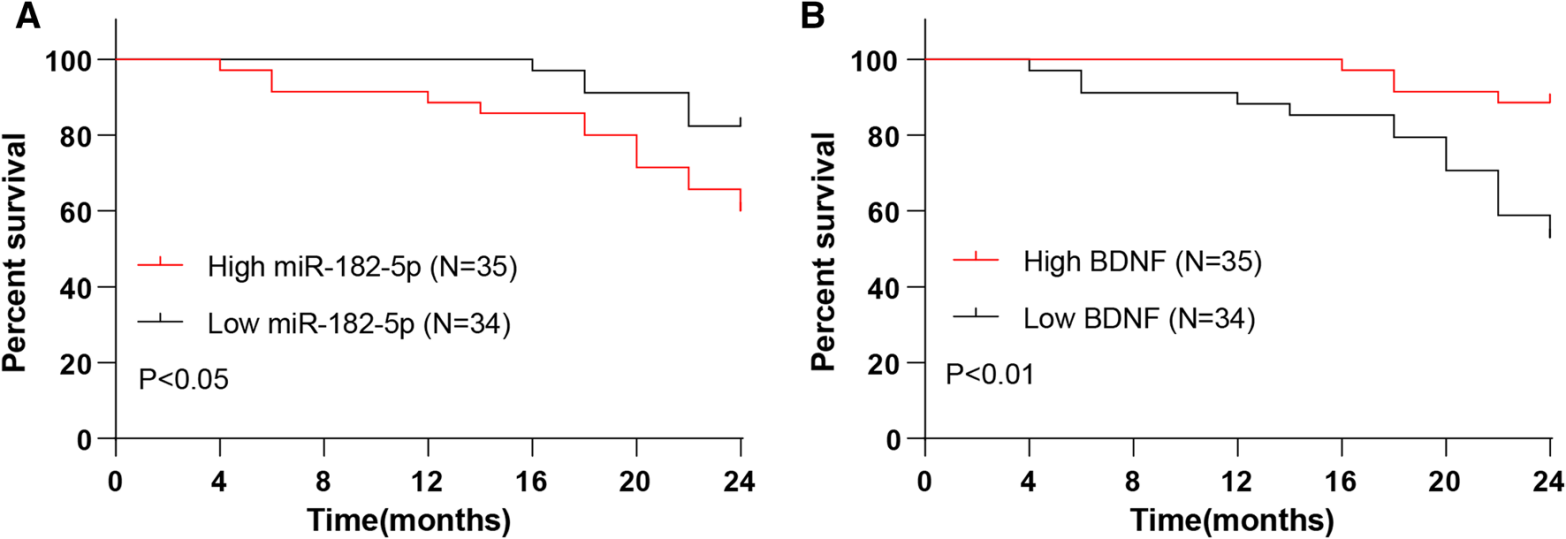
miR-182-5p high expression and BDNF low expression predicted poor prognosis in CHF. Kaplan–Meier curve was used to analyze the survival rate of miR-182-5p/BDNF high expression group and low expression group

To further evaluate the value of miR-182-5p/BDNF in the prognostic evaluation of CHF patients, we conducted regular follow-up for 82 CHF patients for 2 years, collected and analyzed the survival status of all patients, and finally included 69 cases for the prognostic analysis after excluding the lost cases and cases for other causes of death. The CHF patients were divided into miR-182-5p/BDNF high expression group (N = 34) and low expression group (N = 35) according to the median of miR-182-5p/BDNF level. The Kaplan–Meier analysis unveiled that patients in the miR-182-5p low expression group had a higher survival rate than miR-182-5p high expression group, and the BDNF high expression group had an elevated survival rate relative to the BDNF low expression (all *P* < 0.05) (Fig. 5), demonstrating that miR-182-5p high expression and BDNF low expression predicted poor prognosis in CHF.

## Discussion

CHF is a complicated syndrome caused by functional and structural disturbances that impact the heart function in supplying oxygen to tissues [31]. Changes in miRNA level are related to the dysfunctional gene expression profiling in the nosogenesis of HF [32]. As an indicator, BDNF exerts a relatively high predictive value for HF [33]. This study investigated the clinical value of miR-182-5p and BDNF in CHF patients.

miRNAs are involved in various cardiac biological processes and are possible markers for HF [34–36]. miR-182-5p is probably an initial index of cardiac injury and is related to HF [37], and the overexpression of miR-182-5p impacts the onset of arrhythmias and heart morphology [38]. RT-qPCR revealed that miR-182-5p was upregulated in CHF individuals and increased in parallel with the NYHA stage. A report documents that miR-182 is dysregulated in HF patients [39]. Altogether, miR-182-5p engaged in CHF progression. Some miRNAs may regulate the expression of BDNF in different diseases [40]. The administration of recombinant human BDNF improves the exercise ability of HF mice [41]. Hence, we explored the relationship between miR-182-5p and BDNF in CHF. ELISA unveiled the downregulated BDNF in the serum of CHF patients. Similar to our result, the previous study also indicates that the BDNF expression is reduced in CHF patients and regarded as a risk indicator for CHF [42, 43]. The binding sites of miR-182-5p and BDNF were predicted via Starbase database and validated using dual-luciferase assay. Furthermore, Pearson coefficient unraveled that serum miR-182-5p was inversely correlated with BDNF in CHF. It has been supported that BDNF is a new target of the miR-183/96/182 cluster [44, 45]. Altogether, miR-182-5p and BDNF vital players in CHF.

BNP commonly aids the diagnosis, evaluates the therapy effect, and predicts outcomes in HF [46, 47], which is also the predictor for adverse outcomes in CHF [48]. LVEF is the most used measurement index of cardiac systolic function [49, 50]. The analysis of the clinical baseline characteristics illustrated an elevated BNP expression and reduced LVEF level in CHF patients. HF was diagnosed by the typical signs of an enhanced BNP [51] and reduced LVEF levels [52, 53]. Subsequently, the association of serum miR-182-5p/BDNF with BNP and LVEF was assessed using Pearson assay to explore the clinical significance. The results suggested that miR-182-5p was positively associated with BNP and inversely related to LVEF, while the correlation of BDNF with BNP or LVEF was the opposite. Several miRNAs have positive relations with BNP in CHF patients [54, 55]. miRNAs (including miR-182-5p) are linked with LVEF [56]. There exists a positive association between serum BDNF and LVEF [57]. However, there are limited reports about the relationship between serum miR-182-5p/BDNF with BNP and LVEF in CHF. These findings could contribute to the clinical diagnosis of CHF.

miRNA is proposed as a possible diagnostic and prognostic marker in HF [14, 58], and is tightly linked with the curative effect against CHF [59]. Hence, we further evaluated the diagnostic effect of miR-182-5p/BDNF for CHF via the ROC curve. The results illustrated that the AUC of miR-182-5p was 0.780 and the cutoff value was 1.125 (60.89% sensitivity and 84.62% specificity), the AUC of BDNF was 0.829 and the cutoff value was 23.70 (93.90% sensitivity and 64.10% specificity) and the AUC of miR-182-5p combined with BDNF was 0.894 and the cutoff value was 0.665 (71.95% sensitivity and 89.74% specificity). miR-182-5p can assist the diagnosis of arrhythmogenic cardiomyopathy and unprotected left main coronary artery disease [60, 61]. BDNF has a certain diagnostic value in acute HF [62]. The level of plasma BDNF is reduced in HF patients and correlated with HF severity, which can also act as a potential clinical biomarker of HF [63]. Briefly, miR-182-5p combined with BDNF had high diagnostic efficacy for CHF.

Consequently, the role of miR-182-5p/BDNF in the prognostic evaluation of CHF individuals was analyzed by 2-year follow-up after the patients were assigned into miR-182-5p/BDNF high expression and low expression groups. The Kaplan–Meier method demonstrated an increased survival rate in the CHF patients with miR-182-5p low expression or BDNF high expression. miR-182 is capable to predict cardiovascular mortality and is considered as the potential prognostic biomarker in congestive HF [18]. CHF individuals with lowered BDNF experience higher rates of heart events than those with elevated BDNF and low BDNF is associated with reduced survival [28]. Moreover, diminished serum BDNF expression is related to the rehospitalization and death in HF patients, suggesting the involvement in adverse events and poor prognosis [64, 65]. Collectively, upregulated miR-182-5p and downregulated BDNF predicted poor prognosis in CHF.

To summarize, this prospective study initially determined the expression patterns of miR-182-5p in the serum of CHF patients and explored the role of miR-182-5p expression for the diagnosis and classification of CHF by ROC curve. In addition, the effect of miR-182-5p level on CHF was clarified by survival analysis to provide a breakthrough point for the clinical judgment and grading in CHF. However, this study only included elderly CHF patients as subjects, with a short time span, which may have an impact on miR-182-5p level determination. The amount of included samples and events was insufficient. In future studies, we shall conduct a multi-center prospective experiment with the sample size expanded to further ascertain the diagnostic and prognostic evaluation ability of miR-182-5p. Furthermore, serum miR-182-5p level in the early or middle stage of CHF can be determined to assess its diagnostic and prognostic predictive value in the early stage of CHF.

## Data Availability

The data that support the findings of this study are available from the corresponding author upon reasonable request.
